# NAT10 Mediates *XPO1* mRNA N4-acetylation and Promotes Drug Resistance of Myeloma Cells

**DOI:** 10.7150/jca.101403

**Published:** 2024-10-14

**Authors:** Yinyin Xu, Li Wang, Christina He, Zhiqiang Liu, Rong Fu, Ying Xie

**Affiliations:** 1 Tianjin Key Laboratory of Bone Marrow Failure and Malignant Hemopoietic Clone Control; Tianjin Institute of Hematology; Department of Hematology, Tianjin Medical University General Hospital; Tianjin Medical University, Heping, Tianjin 300070 China.; 2 Clinical Laboratory of Yongchuan Hospital, Chongqing Medical University, Chongqing 402160, China.; 3 Tianjin Medical University Cancer Institute and Hospital; National Clinical Research Center for Cancer; Tianjin Key Laboratory of Cancer Prevention and Therapy; Tianjin's Clinical Research Center for Cancer, Tianjin 300192, China.; 4 Carnegie Vanguard High School, Houston, Texas, 77019, USA.

**Keywords:** Multiple myeloma, Chemoresistance, ac4C modification, XPO1, NAT10

## Abstract

The eventually developed chemoresistance to proteasome inhibitors (PIs) is a major hurdle in curing patients with multiple myeloma (MM) and a key cause of poor prognosis, however the underlying molecular mechanisms of chemoresistance is still poorly understood. Herein, we provide evidences that N-acetyltransferase 10 (NAT10), a catalytic enzyme involving in the acetylation modification of RNA, is overexpressed in the BTZ-resistant (BR) MM cell lines and predicts poor outcomes in the clinic. Further manipulating of NAT10 gene expression in MM cells shows that enforced NAT10 expression decreases sensitivity to PI, however knockdown of NAT10 enhances anti-tumor efficacy of PIs in MM cells *in vitro* and* in vivo*. Acetylated RNA immunoprecipitation sequencing (acRIP-seq) combined with RIP-qPCR analysis identifies exportin 1 (XPO1) as an important downstream target of NAT10, with promotes N4-acetylcytidine (ac4C) modification of XPO1 mRNA. Importantly, expressions of XPO1 and NAT10 are meaningfully correlated in bone biopsies from the relapsed/refractory (R/R) MM patients, which were also highly associated with poor outcome. Translationally, dual pharmacological inhibition of NAT10 and XPO1 sensitizes MM cells to BTZ treatment in both cell lines and in a xenograft mouse model of MM. Thus, our study elucidates previously unrecognized role of ac4C modification of XPO1 mRNA in the chemoresistance of MM and provides a potential option for clinical management of R/R MM patients in the clinic.

## Introduction

Multiple myeloma (MM) is characterized by the malignant proliferation of plasma cells in the bone marrow, leading to anemia, cytopenia, fatigue, and bone pain in patients. The global incidence of MM accounts for about 15% of hematological malignancies and 1% of all cancer cases. Despite the development of various treatment modalities such as proteasome inhibitors (PIs), monoclonal antibodies (MoAbs), immunomodulatory drugs, and autologous stem cell transplantation, refractory and relapses remain common, with remission durations progressively shortening. This highlights the importance of understanding the mechanisms involved and developing novel treatment strategies^1^.

Modifications of histone proteins have garnered significant attention due for their regulation of key cancer transcription factors associated with drug resistance^2^. Therapies targeting epigenetic regulators have shown promise in targeting malignant plasma cells post-resistance to initial therapy^3^. For instance, inhibitors of the histone methyltransferase EZH2 have been found to sensitize myeloma cell lines to panobinostat^4^, and when combined with DNA methyltransferases (DNMTs) inhibition, they effectively overcome resistance to IMiDs in myeloma cells^5^. Apart from histone modifications, dysregulation of RNA epigenetics also plays a crucial role in RNA processing, promoting tumor proliferation, metastasis, stemness maintenance, and drug resistance^6^. The enzymes that catalyze or reverse these modifications are considered potential biomarkers and therapeutic targets in cancer^2^, ^7^. For example, m6A is the most abundant mRNA modification, and METTL3, a key component of the m6A methyltransferase complex, promotes the expression of key oncogenes in glioblastoma stem cells^8^, as well as radiotherapy resistance and tumor growth^9^. Moreover, METTL3 is also considered a potential therapeutic target for the treatment of multiple myeloma^7^. These pieces of evidence demonstrate the possibility for overcoming chemoresistance by manipulating epigenetic regulators in MM cells and indicate the urgent need for finding additional epigenetic regulators 10.

Notably, ac4C is the first and only acetylation modification described on mRNA, mainly occurring within coding sequences (CDS), and plays a key role in affecting mRNA stability and translation efficiency^11^. NAT10 is the sole human mRNA ac4C modification enzyme, possessing both acetyltransferase and RNA binding activities^11^. Increasing evidence has shown that abnormal expression of NAT10 promotes the development and prognosis of severe cancer 12, 13. For example, NAT10 promotes malignancy in bladder cancer cells through mediating ac4C modifications in mRNAs^14^. In MM, NAT10 acetylates CEP170 mRNA to accelerate cell growth^15^. The therapeutic value of NAT10 inhibitor Remodelin has been found in many cancers^16^, ^17^. However, whether it epigenetically drives chemoresistance in MM cells is unknown, and its functional targets in MM remain to be clarified.

In the current study, we demonstrate the correlation of NAT10 expression with an established BTZ-resistant MM cells, as well as its significance in the clinic. We identify XPO1 mRNA as a major downstream target of NAT10 and evaluate the translational significance of combination treatment of Remodelin with Selinexor in overcoming bortezomib resistance* in vitro* and* in vivo*.

## Materials and Methods

### Participants and study design

All participants were subjected to a complete history-taking and full clinical examination. All patients have been diagnosed with MM. Patients with a previous diagnosis of MM who received chemotherapy or radiotherapy, cancer anywhere in the body except MM, and any disease in the bone marrow except MM were excluded from the study.

### Cell lines, cell proliferation and flow cytometry assays

MM cell lines used in our lab and the induction of BTZ-resistant cells have been described in previous publication^18^. Cells were STR authenticated and mycoplasma-free substantiated. MST assay to detect cell proliferation and flow cytometry to detect the apoptosis were performed as previously described^18^, ^19^.

### Infection and transfection

Briefly, 1×10^6^ MM cells in 1 ml media were added with 50 μl viral concentration and 8 μg/mL polybrene for 12 h, the medium was changed, and cells were cultured for another 48 h until further management. For transient transfections of plasmids, the Neon transfection system (Invitrogen) was used. Briefly, 1×10^6^ cells were mixed with 10 μg of plasmids and the electroporation was performed under the condition of 1600 V, 20 V, and 1 pulse.

### Immunohistochemistry

The study included MM tissue samples of 40 cases, and tissue samples obtained by bone marrow biopsy. There are 18 samples with low expression of NAT10 and XPO1, and 22 samples with high expression. 40 Paraffin-embedded tissue blocks of MM specimens were prepared by bone marrow biopsy, which were previously fixed with 4% paraformaldehyde (PFA) in PBS for 48 hours. Sections 4 um thick were obtained. 3% H_2_O_2_ solution was used to block deparaffinized tissue slides and a 10 mM citrate buffer (pH 6.0) was used to retrieve antigen. After successful blocking of the deparaffinized tissue, appropriately diluted primary antibodies were added onto the slides and incubated in a humidified chamber at 4ºC overnight, after which diluted biotinylated secondary antibody were incubated for 1 hour at room temperature. DAB substrate solution (Dako, K5361), which was newly made just before use, was utilized to reveal the color of antibody staining. Hematoxylin staining was used to localize Nuclei 1 to 2 min before mounting and capture.

### Establishment of Bortezomib (BTZ)-resistant myeloma cells

To develop bortezomib-resistant (BR) myeloma cells, parental drug-naive cells were initiated by 0.5 nM of BTZ and enhanced by doubled dosage very one month up to six months totally. Acquire of BTZ-resistant phenotype were monitored and confirmed by calculating the IC_50_ of BTZ using MTS assay. For further details on the process of inducing drug resistance please refer to the literature^20^.

### NOD/SCID xenograft mice models

Animal studies were approved by the Committee on Animal Research and Ethics of Tianjin Medical University, and all protocols were confirmed to the Guidelines for Ethical Conduct in the Care and Use of Nonhuman Animals in Research. 4-6 weeks old female NOD.*Cg-Prkdc^scid^Il2rg^tm1Wjl^*/SzJ (NOD/SCID) mice were used to establish the xenograft (n=6) as previously reported^19^, ^21^. For Xenograft model, MM cells (1×10^6^ cells/mouse) were injected subcutaneously into NOD/SCID mice. After 3 weeks, mice were treated subcutaneously with BTZ (1 mg/kg) (n=6) every three days, BTZ (1 mg/kg) + Remodelin (10 mg/kg) (n=6) every three days, BTZ (1 mg/kg) + Selinexor (10 mg/kg) (n=6) every three days or BTZ (1 mg/kg) + Remodelin (10 mg/kg) + Selinexor (10 mg/kg) (n=6) every three days. Mice were weighted and tumors were measured every week. After treatment 8 weeks, for xenografts experiments, mice were sacrificed and the tumor xenografts were collected for TUNEL analysis.

### Ac4C dot blot

Total RNA was heated to 65ºC for 5 min, followed by immediately placed on ice for 1 min, and loaded onto Hybond-N+ membranes. Membranes were crosslinked with 150 mJ/cm2 in a UV254 nm Stratalinker 2400 (Stratagene). Then membranes were blocked with 5% nonfat milk for 1 h at room temperature and incubated with an anti-ac4C antibody (ab252215; Abcam) in PBST (1:1000) at 4℃ overnight. The membranes were then washed three times with 0.1% PBST, incubated with 0.02% methylene blue solution for 10 min, rinsed the background with DEPC water and scanned as an internal reference.

### Acetylated RNA immunoprecipitation and qPCR (acRIP-qPCR)

MM cells were used for acRIP-seq analysis. The procedure of acRIP-seq was performed as previously described^11^. Per each IP, 2.5 mg of anti-NAT10 antibody or 2.5 mg of rabbit IgG control was used. Precipitated RNA was analyzed by qPCR.

### RNA decay assay

MM cells were treated with mRNA transcription inhibitor actinomycin D (5 mg/mL) (HY-17559, MCE) for 0, 1, 2, and 3 h. Then, the total mRNA was isolated and used for RT-qPCR to quantify the relative abundance of DKK1 mRNA (relative to 0 h), and 18 S rRNA was used as internal control.

### 5'-Bromo-uridine (BrU) immunoprecipitation chase-deep RT-qPCR (BRIC RT-qPCR)

Cells were incubated at 37ºC in the presence of 150 μmol/L 5'-bromo-uridine (BrU; 850187, Sigma-Aldrich) for 24 h in a humidified incubator with 5% CO2. The cells were collected at indicated time points after replacing BrU containing medium with BrU-free medium. Total RNA was isolated by using TReasy. BrU-labeled total RNA (12 mg) was denatured by heating at 80ºC for 1 min and then added to the anti-BrdU mAb conjugated beads containing 2 mg of anti-BrdU mAb (MI-11-3, MBL). The mixture was incubated at 4ºC overnight with rotation. After immunoprecipitation, elution of RNA was carried out by adding 300 mL of TReasy directly to the mixture. BrU labeled RNA was extracted and then used for RT-qPCR.

### Statistical analysis

Data were shown as mean ± SEM for at least n=3 independent experiments except otherwise explanation. Differences between groups were determined using paired two-sided Student's *t*-test or one-way ANOVA. Pearson correlation test was used to determine the correlations between gene expressions, and survival analysis and a log-rank test was done by GraphPad Prism 5.0. A* p* value less than 0.05 was considered statistically significant compared with the controls, respectively.

## Results

### NAT10 is highly expressed in the BTZ-resistant MM cells and is associated with poor clinical outcomes

**Figure 1:**
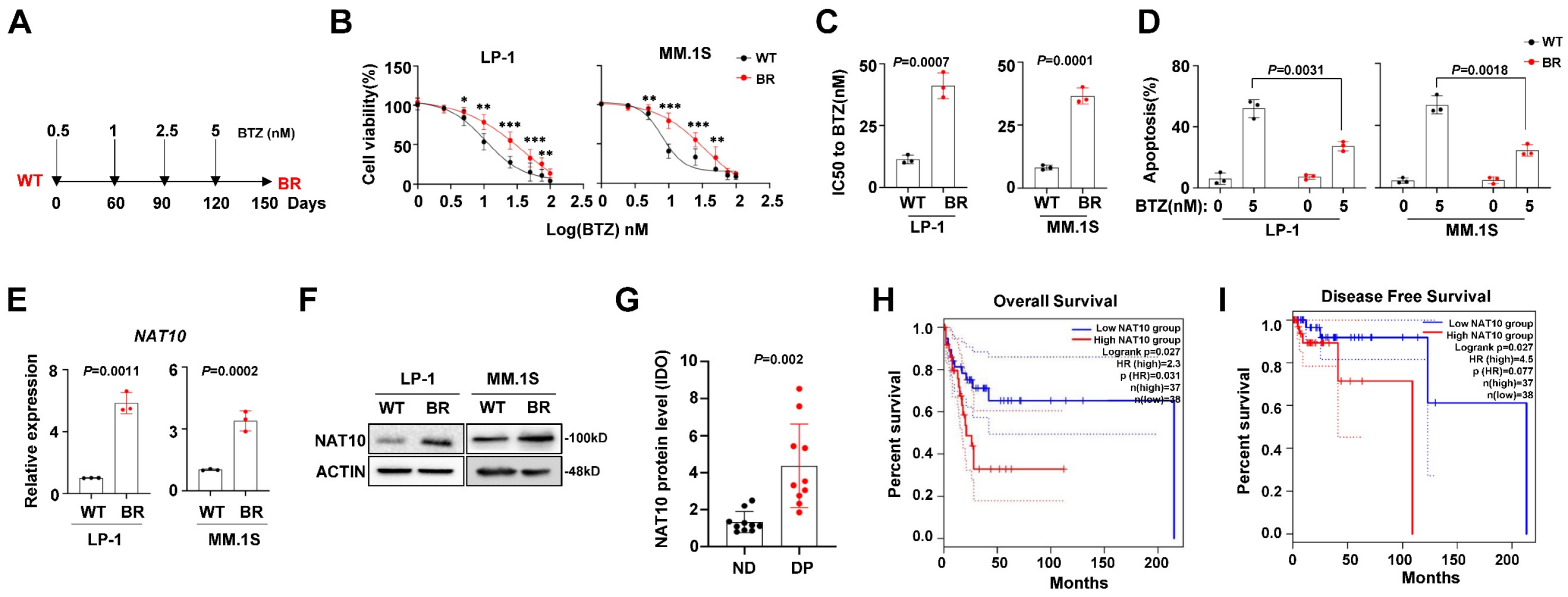
** NAT10 expression is correlated with BTZ resistance in MM cells. (A)** Diagram of induction of BTZ resistance in human MM cell lines. Cells were exposed to increasing concentrations of BTZ (from 0.5 nM to 5 nM) for three months. **(B)** Alteration of IC50 to BTZ treatment in the wild type (WT) and BTZ-resistant (BR) LP-1 and MM.1S cells. **(C)** Comparison of the IC50 values of WT and BR cells (n = 3 biologically independent experiments; mean ± SEM). **(D)** Flow cytometry analysis of apoptosis of WT and BR cells after BTZ treatment. (n =6 biologically independent experiments; mean ± SEM.) **(E)** NAT10 mRNA **(F)** and protein expression in WT and BR LP-1 and MM.1S cells. **(G)** IDO^+^ area measurement of immunohistochemical staining for NAT10 protein in the MM tissue slides from newly diagnosed (ND) patients and patients with disease progression (DP). **(H, I)** Correlation of NAT10 mRNA expression with Overall Survival (OS) and Disease-Free Survival in MM patients after receiving BTZ-based treatment regimens. *p* values were determined by Pearson Coefficient and Log-ranks test. Two-sided *p* values were determined by Student's t test. Data indicate the mean ± SEM.

To investigate the features of BTZ-induced chemoresistance in MM cells, we established two BTZ-resistant (BR) cell lines, LP-1 and MM.1S, following a previously reported method^20^ (**Fig. 1A**). After 3 months of induction, we assessed the sensitivity to BTZ. The fold changes in the inhibitory concentration (IC50) were significantly increased in the BR cells (**Fig. 1B, 1C**). Furthermore, the BTZ-induced apoptosis of MM cells was reduced in the BR MM cells (**Fig. 1D**). It has been reported that NAT10 promotes the proliferation of MM cells^22^, but its role in drug resistance in MM is not clear. Therefore, we evaluated the expression of NAT10 in the BR and wild-type (WT) MM cells. We observed an increase in both protein and mRNA levels in the BR cells (**Fig. 1E, 1F**). but its role in drug resistance in MM is not clear. Therefore, we evaluated the expression of NAT10 in the MM tissue slides from newly diagnosed (ND) patients and patients with disease progression (DP). We observed an increase in protein level in the DP group (**Fig. 1G**). Importantly, higher NAT10 expression was associated with worse overall survival (OS) (**Fig. 1H**) and disease-free survival (**Fig. 1I**). These results suggest that aberrant expression of NAT10 is closely correlated with MM progression.

### NAT10 expression is associated with chemosensitivity of MM cells

**Figure 2:**
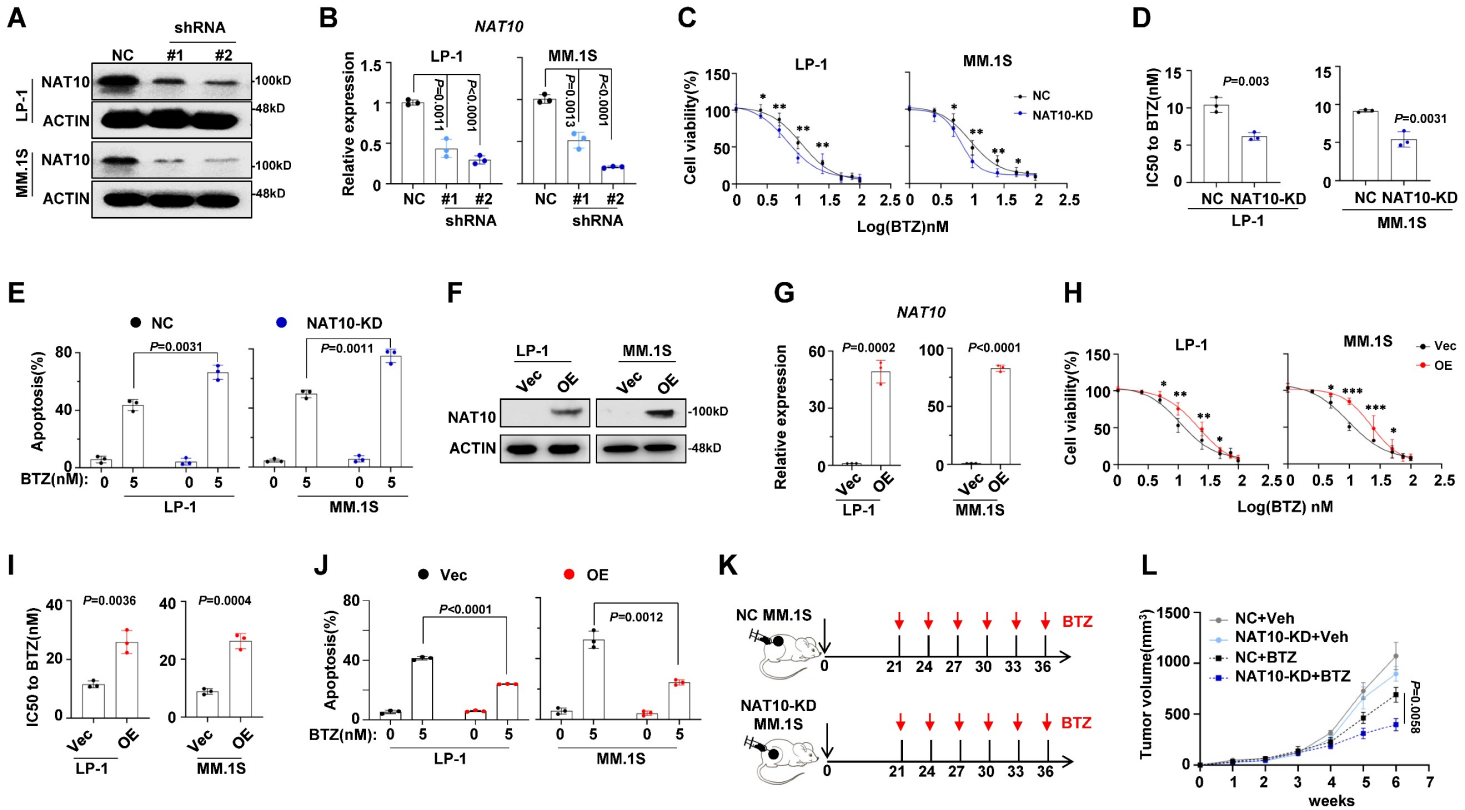
** Manipulation of NAT10 expression alters sensitivity to DTIC treatment *in vitro* and *in vivo*. (A)** Representative western blotting (n = 3 biologically independent experiments) shows the knockdown effects in LP-1 and MM.1S cells infected with lentivirus carrying three shRNAs targeting two different coding sequencing of NAT10 gene (shRNA#1, #2) compared to the non-target control (NC). **(B)** Histogram showing NAT10 relative expression of shNAT10 and NC LP-1 and MM.1S cells. **(C)** Alteration of IC50 to BTZ treatment in the NT Control (NC) and NAT10 knockdown (shNAT10) cells and **(D)** comparison of the IC50 values of NC and shNAT10 cells (n = 3 biologically independent experiments). Two-sided *p*-values were determined by Student's t test; mean ± SEM.** (E)** Frequency of apoptosis cells after BTZ treatment.** (F)** Representative western blot (n=3 biologically independent experiments) shows the ectopic expression of NAT10 in LP-1 and MM.1S cells infected with lentivirus carrying the NAT10- overexpression plasmids (NAT10- OE) compared to the vector control (Vec).** (G)** Histogram showing NAT10 relative expression of NAT10- OE and Vec LP-1 and MM.1S cells. **(H)** Alteration of IC50 to BTZ treatment in the Vec and NAT10- OE cells and **(I)** comparison of the IC50 values of Vec and NAT10- OE cells (n = 3 biologically independent experiments). **(J)** Frequency of apoptosis cells after BTZ treatment. **(K)** Experimental setup used to assess the effects of NAT10 on MM tumor growth and BTZ sensitivity *in vivo* in NOD/SCID mice. Mice were subcutaneous (s.c.) injected with 1 × 10^6^ NC or NAT10- KD LP-1 cells followed by i.v. injections of BTZ (1 mg/kg) or PBS every three days (n=6). Tumor growth was measured and calculated as 1/2(L × W^2^) mm, where the L presenting the length and W representing width of tumor. **(L)** Relative tumor growth curves of tumors. Two-sided *p*-values were determined by two-way ANOVA test; mean ± SEM.

To further investigate the impact of NAT10 on drug resistance in MM cells, we knocked down NAT10 expression using short hairpin RNA (shRNA). As expected, the expression levels of NAT10 in LP-1 and MM.1S cells were successfully reduced by shRNAs (**Fig. 2A, 2B**), leading to a significant decrease in IC50 values for BTZ (**Fig. 2C, 2D**) and impaired anti-apoptotic capacity (**Fig. 2E**). Conversely, overexpression of NAT10 in LP-1 and MM.1S cells **(Fig. 2F, 2G)** significantly increased the IC50 value (**Fig. 2H, 2I**) and inhibited apoptosis in MM cells treated with BTZ (**Fig. 2J**). To further evaluate the effects of NAT10 on myeloma tumor growth and BTZ sensitivity *in vivo*, we established a xenograft model of MM by subcutaneously injecting NAT10-KD and negative control (NC) MM cells in NOD/SCID mice, and then administered BTZ or vehicle control intravenously every three days (**Fig. 2K**). We observed that mice bearing NAT10-KD and NC MM cells had smaller tumor growth, however administration of BTZ remarkably suppressed tumor growth derived from NAT10-KD cells than that from NC controls **(Fig. 2L).** These findings collectively suggest that NAT10 plays a role in the chemosensitivity of MM cells both *in vitro* and *in vivo*.

### NAT10 mediated ac4C modification enhances XPO1 mRNA stability and translation efficiency

**Figure 3:**
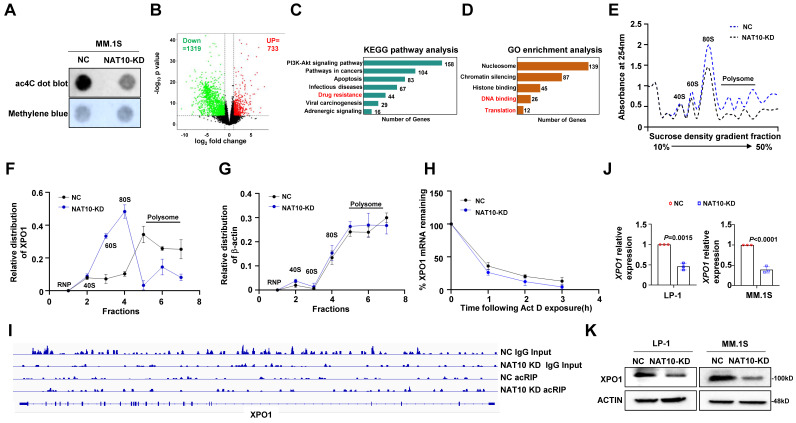
** NAT10 acetylates XPO1 mRNA to enhance translation efficiency. (A)** Representative anti-ac4C dot blot performed on total RNA of NAT10-KD and NC cells. **(B)** Volcano map of differentially expressed ac4C gene peaks upon NAT10 knockdown.** (C)** KEGG pathway enrichment analysis of acRIP-seq. KEGG, Kyoto Encyclopedia of Genes and Genomes. **(D)** GO enrichment analysis of acRIP-seq. GO, Gene Ontology. **(E)** Polysome profile assay shows an overall decreased tendency of translation efficiency in NAT10-KD MM cells.** (F, G)** qPCR showing the changed relative distribution of NAT10 mRNA in different polysome gradient fractions between control and NAT10-KD cells. β-actin without ac4C modification is used as control mRNA. **(H)** BRIC RT-qPCR assay measuring the half-life of DKK1 mRNA in NAT10- KD and NC MM cells (n= 3). **(I)** Gene tracks showing representative acRIP-Seq profiles at XPO1 gene loci in NAT10-KD and NC LP-1 cells. **(J)** XPO1 mRNA expression and **(K)** protein level in NAT10-KD and NC MM cells. n = 3, *p* value determined by Student's *t* test.

Given that NAT10 functions as an acetyltransferase, we investigated whether NAT10-mediated chemoresistance in MM cells is dependent on its acetyltransferase activity. We assessed mRNA acetylation levels in MM cells using a dot blot assay and observed that NAT10 knockdown resulted in decreased total ac4C levels in mRNAs **(Fig. 3A)**. Subsequently, we conducted acRIP-seq assays in NAT10-knockdown and control LP-1 cells, identifying 1319 genes with reduced ac4C modification in NAT10-knockdown cells compared to the control cells **(Fig. 3B)**. Analysis of KEGG pathway showed that the genes associated with drug resistance was enriched among the downregulated differentially expressed genes (DEGs) upon NAT10 knockdown **(Figure 3C)**. According to GO analysis, downregulated DEGs were typically enriched in DNA binding and translation **(Figure 3D)**. Additionally, NAT10 knockdown significantly reduced translation efficiency, as demonstrated by polysome fractionation assays (**Fig. 3E**). We noted that XPO1 mRNA was among the top targets of NAT10, prompting us to perform RT-qPCR to analyze the distribution of XPO1 in different ribosome fractions. In NAT10-knockdown cells, XPO1 mRNA was predominantly found in monosomes or light polysomes, whereas in control cells, it was mainly detected in polysomes, indicating a role of ac4C in XPO1 translational regulation (**Fig. 3F, 3G**). RNA decay assays revealed lower stability of XPO1 transcripts in NAT10-knockdown cells compared to control cells** (Fig. 3H)**. The acRIP data showed a significant decrease in ac4C peaks of XPO1 mRNA upon NAT10 knockdown **(Fig. 3I)**. Furthermore, both protein expression and RNA levels of XPO1 were substantially reduced in NAT10-knockdown cells (**Fig. 3J, 3K**). These findings collectively suggest that NAT10 promotes XPO1 accumulation in MM cells by acetylating XPO1 mRNA to enhance its translation.

### XPO1 is correlates with NAT10 expression and MM progression

**Figure 4:**
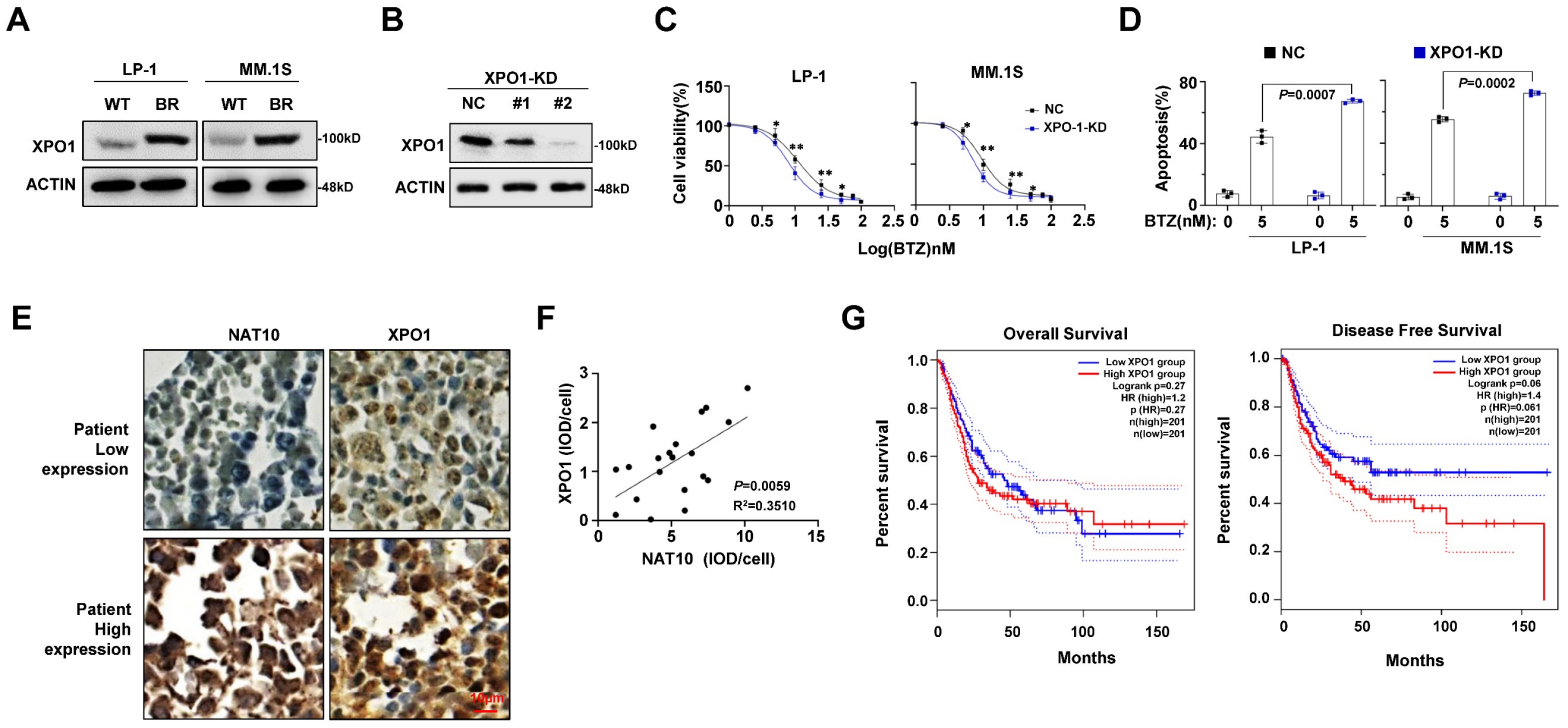
** XPO1 expression correlated with MM malignancy. (A)** Protein level of XPO1 after induction of BTZ resistance in LP-1 and MM.1S cells.** (B)** Representative Western blotting shows the knockdown effects in LP-1 cells infected with lentivirus carrying two shRNAs targeting different coding sequencing of XPO1 gene (shRNA#1, #2) compared to the non-target control (NC).** (C)** Alteration of IC50 to BTZ treatment in the NC and XPO1 knockdown (KD) cells.** (D)** Flow cytometry analysis of cell apoptosis induced by 5nM BTZ for 24 h. **(E)** Representative immunohistochemical staining for NAT10 and XPO1 protein in MM issue slides from same patient show the correlation of expression. Scale bar: 10µm.** (F)** Correlation of NAT10 with XPO1 expression in clinical samples of MM patients (n=20). **(G)** Correlation of XPO1 expression with disease-free survival and OS in MM patients after receiving BTZ -based treatment regimens. All *p*-values were determined by Pearson Coefficient and Log-ranks test. Data represent mean± SEM.

Since XPO1 is downstream target of NAT10, we next determined its role in BTZ sensitivity. Indeed, increased XPO1 expression were also found in our established BR MM cells (**Fig. 4A**), and when the expression of XPO1 was successfully suppressed (**Fig. 4B**), the sensitivity to BTZ were largely improved, as evidenced by lower IC50 value (**Fig. 4C**) and markedly augmented apoptotic cells (**Fig. 4D**). Immunohistochemical staining of NAT10 and XPO1 proteins in MM patient tissues showed a close correlation in their expressions (**Fig. 4E, 4F**). Further analysis revealed a negative correlation between XPO1 expression and both disease-free survival and overall survival of MM patients following BTZ-based treatments (**Fig. 4G**). These results suggest that XPO1 expression is associated with NAT10 expression and significantly impacts treatment response and clinical outcomes in MM.

### Combinational inhibition of NAT10 and XPO1 sensitizes MM cells to bortezomib treatment *in vitro* and *in vivo*

**Figure 5:**
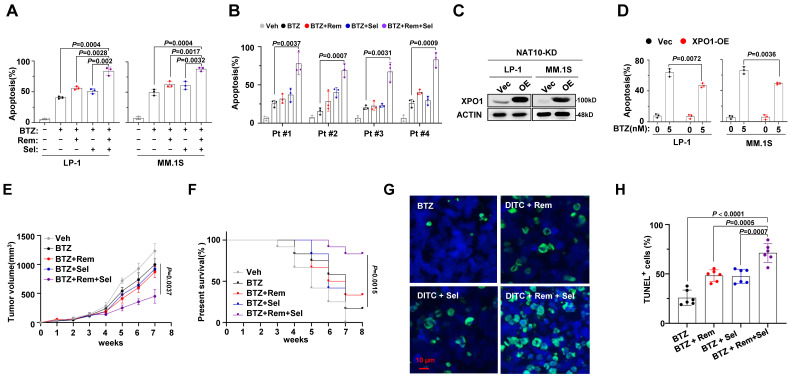
** Effect of combinational treatment with Remodelin and Selinexor in sensitizing MM cells to bortezomib *in vitro* and *in vivo*. (A)** Flow cytometry analysis for apoptosis of MM cell lines or** (B)** CD138^+^ plasma cells from MM patients treated with BTZ (5nM) in presence of Remodelin (300nM) and Selinexor (300nM) for 48 h. *p* value determined by Student's *t* test for n= 3 independent experiments. **(C)** Western blotting shows the ectopically expression of XPO1 in NAT10-KD LP-1 and MM.1S cells.** (D)** Frequency of apoptosis cells after BTZ treatment. **(E)** 1×10^6^ BR LP-1 cells were used to establish xenograft MM model in NOD/SCID mice, growth of tumors in mice receiving DMSO (Veh), BTZ (1 mg/kg), BTZ plus Remodelin (10 mg/kg) or Selinexor (10 mg/kg), and combination treatment of Remodelin and Selinexor plus BTZ were measured every week (n= 6/group). Differences between groups were analyzed using one-way ANOVA. **(F)** Kaplan-Meier curves showing survival of mice. **(G)** Confocal image of representative immunofluorescence staining for tumor tissue TUNEL (Alexa Fluor 488, green) and nuclei (DAPI, blue) at 4 weeks. **(H)** Quantification of TUNEL-positive cells. Two-sided *p*-values were determined by two-way ANOVA test; mean ± SEM.

To elucidate the translational implications of our findings, we investigated whether a synergistic anti-MM effect could be achieved by combining small molecules targeting NAT10 (Remodelin) and XPO1 (Selinexor) with BTZ. While Remodelin and Selinexor alone did not sensitize MM cells to BTZ, their combination significantly enhanced the anti-MM efficacy of BTZ (**Fig. 5A**). Importantly, this synergistic effect was also observed in CD138+ plasma cells isolated from MM patients with disease progression after BTZ-based regimens (**Fig. 5B**). Restoring XPO1 expression in NAT10-knockdown MM cells partially reversed resistance to BTZ treatment (**Fig. 5C, 5D**).

To further evaluate the efficacy of combined treatment with Remodelin and Selinexor in overcoming BTZ resistance, we established a xenograft mouse model of multiple myeloma (MM) using our previously established BR-LP-1 cells. Despite BR-LP-1 cells showing resistance to BTZ treatment alone, the combination of either inhibitor resulted in improved anti-tumor effects. Moreover, a synergistic effect was observed when BTZ was combined with both inhibitors, as evidenced by reduced tumor volume (**Fig. 5E**) and increased survival rate of mice (**Fig. 5F**). TUNEL assay confirmed that the combined treatment with Remodelin and Selinexor induced significant cell apoptosis in the tumors (**Fig. 5G, 5H**). These findings strongly suggest that targeting NAT10 and XPO1 could re-sensitize resistant MM cells to BTZ treatment and enhance the anti-MM effects of proteasome inhibitors.

## Discussion

Bortezomib, approved by the FDA as a first-line agent for MM treatment, faces challenges due to the development of chemoresistance. Our study reveals the critical role of NAT10 in inducing drug resistance by mediating acetylation of XPO1 to promote its translation and expression. We demonstrate the synergistic anti-MM effects of the NAT10-specific inhibitor Remodelin and the XPO1 inhibitor Selinexor, offering potential strategies for refractory and relapsed MM patients.

Mounting evidence suggests that dysregulation of mRNA modifications drives cancer progression^23^. As the sole enzyme NAT10 catalyzes of RNA ac4C, its role in hematologic malignances drug resistance, especially in MM, is unknown. We identify increased NAT10 in the resistant MM cells, which correlates with PIs sensitivity and clinical outcomes. NAT10 was found to participate serious critical cell processes as a acetyltransferase of protein, for example, it responses to stress via acetylating and activating p53^24^, regulates cytokinesis through acetylation of microtubules^25^, and activates rRNA transcription through autoacetylation. We elucidate that NAT10 promotes MM drug resistance through RNA acetyltransferase activity, with XPO1 identified as its main downstream target in inducing chemoresistance. XPO1 is a nuclear export receptor, key tumor suppressor proteins, including RB, p53, and p21 were found cargo of XPO1 transported^26^. XPO1 overexpression is a common feature among many human cancer types. In MM, it is associated with poor prognosis^27^, progression from monoclonal gammopathy of undetermined significance to active multiple myeloma^27^, and XPO1 was thought to be potentially biomarker of cancer progression as well as development of drug resistance in MM^28^. XPO1 inhibitors Selinexor have been demonstrated to exert antitumor activity in a broad range of cancer types including MM^28^, while the mechanism of XPO1 overexpression remains unknown. We examined the mRNA-acetyltransferase activity of NAT10 in MM cells by acRIP-seq, and identified XPO1 is a major downstream target of NAT10. Our RNA decay assay and polysome profile assay demonstrate the role of NAT10 in XPO1 stability and translational efficiency. Consistently, in the NAT10-KD MM cells, decreased ac4c modification were observed, and more specifically, only the protein level of XPO1, but not its mRNA expression was decreased according to NAT10 knockdown. These data highlight the role of NAT10 in mediating ac4c modification in promoting XPO1 mRNA translation, which is consistent with a previous study^11^, ^22^. Our findings highlight the role of NAT10 in promoting XPO1 mRNA translation, shedding light on the mechanism of MM chemoresistance.

Drug resistance is the major obstacle to cure MM in the clinic. Identifying therapy-specific resistance biomarkers is also of great value. XPO1 inhibition is a mechanistically unique strategy compared to the current therapy, so it may has the potential to overcome drug resistance^28^, ^29^. Our study underscores the synergistic efficacy of the NAT10 inhibitor Remodelin and XPO1 inhibitor Selinexor in overcoming BTZ resistance. Administration of Selinexor and Remodelin alongside BTZ efficiently induces tumor cell death in MM cell lines, patient CD138+ plasma cells, and mouse xenograft models, suggesting a promising approach for treating relapsed or refractory MM patients.

## Conclusion

In summary, our study discloses the importance of NAT10 in inducing drug resistance of MM cells, and elucidates the mechanism of NAT10 in mediating ac4c modification of XPO1 mRNA. Our findings provide insights for the development of novel therapeutic strategies utilizing Remodelin and Selinexor for MM patients who have relapsed or become refractory to proteasome inhibitor treatment.

## Abbreviations

PIs: proteasome inhibitors
MM: multiple myeloma
NAT10: N-acetyltransferase 10
BTZ: Bortezomib
BR: BTZ-resistant
acRIP-seq: Acetylated RNA immunoprecipitation sequencing
XPO1: exportin 1
ac4C: N4-acetylcytidine
R/R: relapsed/refractory
MoAbs: monoclonal antibodies
CDS: coding sequences
NOD/SCID: NOD.Cg-PrkdcscidIl2rgtm1Wjl/SzJ
BrU: 5'-Bromo-uridine
IC50: inhibitory concentration
ND: newly diagnosed
DP: disease progression
OS: overall survival
shRNA: short hairpin RNA
DEGs: differentially expressed genes

## References

[1] Siegel RL, Miller KD, Jemal A (2018). Cancer statistics, 2018. CA Cancer J Clin.

[2] Barbieri I, Kouzarides T (2020). Role of RNA modifications in cancer. Nature reviews Cancer.

[3] Yang T, Liu X, Kumar SK, Jin F, Dai Y (2022). Decoding DNA methylation in epigenetics of multiple myeloma. Blood reviews.

[4] Harding T, Swanson J, Van Ness B (2018). EZH2 inhibitors sensitize myeloma cell lines to panobinostat resulting in unique combinatorial transcriptomic changes. Oncotarget.

[5] Dimopoulos K, Sogaard Helbo A, Fibiger Munch-Petersen H, Sjo L, Christensen J, Sommer Kristensen L (2018). Dual inhibition of DNMTs and EZH2 can overcome both intrinsic and acquired resistance of myeloma cells to IMiDs in a cereblon-independent manner. Molecular oncology.

[6] Kumari S, Kumar S, Muthuswamy S (2023). RNA N6-methyladenosine modification in regulating cancer stem cells and tumor immune microenvironment and its implication for cancer therapy. Journal of cancer research and clinical oncology.

[7] Bach C, Leffler M, Flamann C, Kronke J, Mougiakakos D, Mackensen A (2018). Role of N6-Methyladenosine (m6A) RNA Modification in Multiple Myeloma. Blood.

[8] Lin S, Choe J, Du P, Triboulet R, Gregory RI (2016). The m(6)A Methyltransferase METTL3 Promotes Translation in Human Cancer Cells. Molecular cell.

[9] Visvanathan A, Patil V, Arora A, Hegde AS, Arivazhagan A, Santosh V (2018). Essential role of METTL3-mediated m(6)A modification in glioma stem-like cells maintenance and radioresistance. Oncogene.

[10] Lemos A, Leao M, Soares J, Palmeira A, Pinto M, Saraiva L (2016). Medicinal Chemistry Strategies to Disrupt the p53-MDM2/MDMX Interaction. Medicinal research reviews.

[11] Arango D, Sturgill D, Alhusaini N, Dillman AA, Sweet TJ, Hanson G (2018). Acetylation of Cytidine in mRNA Promotes Translation Efficiency. Cell.

[12] Jin G, Xu M, Zou M, Duan S (2020). The Processing, Gene Regulation, Biological Functions, and Clinical Relevance of N4-Acetylcytidine on RNA: A Systematic Review. Molecular therapy Nucleic acids.

[13] Cai S, Liu X, Zhang C, Xing B, Du X (2017). Autoacetylation of NAT10 is critical for its function in rRNA transcription activation. Biochemical and biophysical research communications.

[14] Wang GP, Zhang M, Zhang YM, Xie YQ, Zou JP, Zhong JY (2022). NAT10-mediated mRNA N4-acetylcytidine modification promotes bladder cancer progression. Clin Transl Med.

[15] Wei RF, Cui X, Min J, Lin ZG, Zhou YY, Guo MJ (2022). NAT10 promotes cell proliferation by acetylating CEP170 mRNA to enhance translation efficiency in multiple myeloma. Acta Pharm Sin B.

[16] Dalhat MH, Mohammed MRS, Ahmad A, Khan MI, Choudhry H (2021). Remodelin, a N-acetyltransferase 10 (NAT10) inhibitor, alters mitochondrial lipid metabolism in cancer cells. Journal of cellular biochemistry.

[17] Ma N, Liu H, Wu Y, Yao M, Zhang B (2022). Inhibition of N-Acetyltransferase 10 Suppresses the Progression of Prostate Cancer through Regulation of DNA Replication. International journal of molecular sciences.

[18] Xie Y, Liu J, Jiang H, Wang J, Li X, Wang J (2019). Proteasome inhibitor induced SIRT1 deacetylates GLI2 to enhance hedgehog signaling activity and drug resistance in multiple myeloma. Oncogene.

[19] Liu Z, Xu J, He J, Zheng Y, Li H, Lu Y (2014). A critical role of autocrine sonic hedgehog signaling in human CD138+ myeloma cell survival and drug resistance. Blood.

[20] Xie Y, Liu J, Jiang H, Wang J, Li X, Wang J (2020). Proteasome inhibitor induced SIRT1 deacetylates GLI2 to enhance hedgehog signaling activity and drug resistance in multiple myeloma. Oncogene.

[21] Liu H, Liu Z, Du J, He J, Lin P, Amini B (2016). Thymidine phosphorylase exerts complex effects on bone resorption and formation in myeloma. Science translational medicine.

[22] Wei R, Cui X, Min J, Lin Z, Zhou Y, Guo M (2022). NAT10 promotes cell proliferation by acetylating CEP170 mRNA to enhance translation efficiency in multiple myeloma. Acta pharmaceutica Sinica B.

[23] Wang N, Ma T, Yu B (2023). Targeting epigenetic regulators to overcome drug resistance in cancers. Signal Transduct Target Ther.

[24] Liu X, Tan Y, Zhang C, Zhang Y, Zhang L, Ren P (2016). NAT10 regulates p53 activation through acetylating p53 at K120 and ubiquitinating Mdm2. EMBO reports.

[25] Shen Q, Zheng X, McNutt MA, Guang L, Sun Y, Wang J (2009). NAT10, a nucleolar protein, localizes to the midbody and regulates cytokinesis and acetylation of microtubules. Experimental cell research.

[26] Azmi AS, Uddin MH, Mohammad RM (2021). The nuclear export protein XPO1 - from biology to targeted therapy. Nat Rev Clin Oncol.

[27] Schmidt J, Braggio E, Kortuem KM, Egan JB, Zhu YX, Xin CS (2013). Genome-wide studies in multiple myeloma identify XPO1/CRM1 as a critical target validated using the selective nuclear export inhibitor KPT-276. Leukemia.

[28] Muz B, Azab F, de la Puente P, Landesman Y, Azab AK (2017). Selinexor Overcomes Hypoxia-Induced Drug Resistance in Multiple Myeloma. Translational oncology.

[29] Jakubowiak AJ, Jasielec JK, Rosenbaum CA, Cole CE, Chari A, Mikhael J (2019). Phase 1 study of selinexor plus carfilzomib and dexamethasone for the treatment of relapsed/refractory multiple myeloma. British journal of haematology.

